# Under-attending free antenatal care is associated with adverse pregnancy outcomes

**DOI:** 10.1186/1471-2458-7-268

**Published:** 2007-09-27

**Authors:** Kaisa Raatikainen, Nonna Heiskanen, Seppo Heinonen

**Affiliations:** 1 Department of Obstetrics and Gynecology, PO Box 1777, Kuopio University Hospital, 70211 Kuopio, Finland

## Abstract

**Background:**

Most pertinent studies of inadequate antenatal care concentrate on the risk profile of women booking late or not booking at all to antenatal care. The objective of this study was to assess the outcome of pregnancies when free and easily accessible antenatal care has been either totally lacking or low in number of visits.

**Methods:**

This is a hospital register based cohort study of pregnancies treated in Kuopio University Hospital, Finland, in 1989 – 2001. Pregnancy outcomes of women having low numbers (1–5) of antenatal care visits (*n *= 207) and no antenatal care visits (*n *= 270) were compared with women having 6–18 antenatal visits (*n *= 23137). Main outcome measures were: Low birth weight, fetal death, neonatal death. Adverse pregnancy outcomes were controlled for confounding factors (adjusted odds ratios, OR: s) in multiple logistic regression models.

**Results:**

Of the analyzed pregnant population, 1.0% had no antenatal care visits and 0.77% had 1–5 visits. Under- or non-attendance associated with social and health behavioral risk factors: unmarried status, lower educational level, young maternal age, smoking and alcohol use. Chorio-amnionitis or placental abruptions were more common complications of pregnancies of women avoiding antenatal care, and pregnancy outcome was impaired. After logistic regression analyses, controlling for confounding, there were significantly more low birth weight infants in under- and non-attenders (OR:s with 95% CI:s: 9.18 (6.65–12.68) and 5.46 (3.90–7.65), respectively) more fetal deaths (OR:s 12.05 (5.95–24.40) and 5.19 (2.04–13.22), respectively) and more neonatal deaths (OR:s 10.03 (3.85–26.13) and 8.66 (3.59–20.86), respectively).

**Conclusion:**

Even when birth takes place in hospital, non- or under-attendance at antenatal care carries a substantially elevated risk of severe adverse pregnancy outcome. Underlying adverse health behavior and possible abuse indicate close surveillance of the newborn.

## Background

The optimal amount and content of antenatal care in either low- or high-risk pregnancies is not yet resolved. There is, however, evidence showing some unquestionable benefits of antenatal care [1-5] and overall, the outcome of pregnancies among women giving birth at home and without any antenatal care for religious reasons is known to be severely impaired in the US [6]. On the other hand, the results of a recent systematic review suggested that women with low-risk pregnancies can safely have fewer antenatal care visits [2]. Child-bearing women's own expectations are diverse, some wishing for more and some, specifically women over 35 years of age or with an unfortunate timing of pregnancy, wanting fewer antenatal care visits [7].

Most pertinent studies in this field concentrating on the risk profile of women booking late or not booking at all to antenatal care have shown that the most common barriers to attendance at antenatal care in modern Western society are lack of insurance, low income, low educational level, low social class, unmarried status, ethnic origin of the woman, difficulties in obtaining appointments and long distances [8,9].

In Finland, almost the entire (99.8%) pregnant population attends antenatal care [10] since it is provided by the state free of charge and is easily accessible. Furthermore, the attendance is encouraged by linking the opportunity to receive maternity benefits to the first visit to maternity care units before the 16^th ^week of pregnancy. The average number of 17 antenatal care visits is high [11]. This number exceeds both international standards [2] and national recommendations of 13–17 visits in first pregnancy and 9–13 visits in others, two of the visits scheduled after birth [12]. Currently, maternal and perinatal mortality rates [13] and the incidence of suboptimal care are very low [14]. Although the content and frequency of antenatal care are thorough considered, a small minority of women do not attend. The purpose of this study was to assess the outcome of pregnancies when antenatal care has been inadequate – either totally lacking or low in number of visits.

## Methods

We investigated an existing clinical database of the total population of 27776 births at Kuopio University Hospital between 1989 and 2001. Information on maternal characteristics was based on data from self-administered questionnaires at approximately 20 weeks of pregnancy, returned to the hospital by 22 weeks of pregnancy. When any data were missing, they were complemented by interviews with a nurse at the delivery ward. The questionnaire consisted of 75 items, concerning marital status, employment, previous operations, illnesses, obstetric history, contraceptive use, smoking, alcohol consumption and paternal characteristics. Information on pregnancy complications, pregnancy outcome and the neonatal period was collected real timely as part of clinical work to the database by the nurses and midwives who took care of delivery and neonatal care. The Institutional Review Board accepted the study and childbearing women gave informed consent at the time of data collection. The ethical committee has accepted the database and given permission for using it for research purposes. The data were processed anonymously.

Exclusion criteria were: 1) multiple pregnancies (*n *= 548) and 2) major fetal structural anomalies (*n *= 275), because such pregnancies carry an unusually high risk of adverse outcome. Information of the number of antenatal care visits was missing from 77 women. Women who had an extensive number of 19 visits or over to antenatal care units, were considered a clinically different group, with high morbidity, and were not included in this study. We analyzed 23 614 births, of which 270 were among women who did not attend antenatal care (non-attenders) and 207 were among women with few antenatal care visits (1–5, under-attenders). Women with an average number (6–18) of antenatal care visits, totaling 23137, were used as reference group.

Kuopio University Hospital is a tertiary level obstetric referral centre, but it also serves as the only hospital in our district dealing with deliveries. The antenatal care model was general practitioner- and nurse- or midwife-led for women with uncomplicated pregnancies. The appointments were structured and involved clear referral pathways to university hospital obstetricians when complications arose. Antenatal care was readily and easily accessible to all women. Intrapartum care was obstetrician-led and planned home deliveries were exceptional. The women carried their own maternity case notes, and structured, national maternity records were collected.

The following definitions were used: Young maternal age was defined as age under 18 years at birth. Women aged over 35 years at birth were considered old parturients. Unmarried status was defined as any civilian status other than marriage (including cohabiting, single, widowed and divorced women). Unemployment status was asked in the questionnaire *yes/no*. Educational attainment was divided into three categories: high, average and low, according to the women's own evaluation. Grand multiparity was defined as more than 7 previous deliveries. Prior induced abortion of a viable fetus was separated from miscarriage. An overweight condition was considered when pre-pregnancy body mass index (BMI) was over 25 kg/m^2^. The woman was considered a smoker if she smoked 5 cigarettes or more per day during pregnancy. Alcohol use was recorded as *yes/no*. Chronic illnesses were defined as conditions requiring regular medication that has possible effect on pregnancy, specifically, thyroid disease, arthritis, epilepsy, cardio-vascular and kidney diseases. Maternal diabetes was defined as insulin-treatment during pregnancy. Chronic hypertension was self reported as a multiple choice concerning maternal illnesses in the questionnaire. Illness data were complemented by information from the women's maternity case notes that they carry with them, and by clinical records of Kuopio University Hospital. Gestational age of 42+0 weeks or more was used as definition for prolonged pregnancies. Low hemoglobin was defined as under 100 g/l in the third trimester of pregnancy. Pre-eclampsia was defined as repeated blood pressure measurement exceeding 149/90 mmHg with proteinuria exceeding 0.5 g/day. Chorio-amnionitis, placental abruption or placenta previa were registered when these obstetric diagnoses were set during the hospital stay. Abnormal CTG was recorded to the database by obstetricians. Meconium staining of amniotic fluid during delivery was marked in the delivery reports and to the database by midwives.

Preterm birth was delivery before 37 weeks of gestation. Estimation of gestational age was based on menstrual history and ascertained by measuring fetal crown-rump length by ultrasonography in approximately 95% of cases at 10 to 12 weeks of pregnancy. Infants were considered small for gestational age (SGA) when the age- and sex-adjusted birth weight was below the tenth percentile according to the normal tables for our population [15], and of low birth weight (LBW) when it was less than 2500 g. The mode of delivery was registered to the database as: spontaneous, instrumental or cesarean section. Apgar scores were given mainly by midwifes in uncomplicated deliveries and by pediatricians, when consulted, and were considered low when the scores were less than 7. The pH limit used for fetal acidosis was 7.15 at birth in the umbilical vein. Abnormal CTG was recorded to the database by obstetricians. The admission rate to the neonatal intensive care unit (NICU) was recorded as infants requiring more than 24 hours surveillance. Neonates needing only observation are also treated in the NICU in our hospital.

Fetal death was defined as intrauterine death of a fetus over 22 weeks of gestational age or over 500 g weight and neonatal death as death during the first seven days after birth. If a subject had two abnormalities, such as LBW and preterm delivery, each was considered an independent outcome and the subject was included in both categories.

The validity of the data has manually been checked for some specific pregnancy complications, such as perinatal deaths, velamentous umbilical insertions, umbilical cord knots and placental abruptions.

Statistical differences between the subjects and the reference group were evaluated by using Chi-square tests (dichotomous variables), and Fisher's exact test was applied when the minimal estimated expected value was less than five. A value of *p *< 0.05 was considered statistically significant. Continuous variables were analyzed by using two-tailed, pooled *t *tests. Logistic regression analysis controlling for all clinically significant possible confounding factors was performed (SAS for Windows, SAS release 8 statistical package). The logistic regression models included: age under 18 y, age over 35 y, unmarried status, smoking during pregnancy, using alcohol during pregnancy, educational level, primiparity, multiparity, prolonged gravidity, chronic illness and diabetes. Confidence intervals were evaluated at 95%.

## Results

Of the obstetric population of singletons without major anomalies, 270 (1.0%) had no antenatal care visits, 207 (0.77%) had 1–5 visits, and 23137 (85.8%) had an average number (6–18) of antenatal care visits. The differences between the study groups and the reference group in incidence of birth outside hospital were statistically significant (P < 0.025 for under-attenders and P < 0.001 for non-attenders), although the numbers were very small: In the group of 6–18 antenatal care visits 14 (0.09%) infants were born outside hospital, in the group of no antenatal care there were 5 (1.85%) and in the group of 1–5 visits one (0.97%). No difference in ages of the women in the study groups and in the reference group was found: the mean age (± standard deviation) of the women who had 6–18 antenatal visits being 28.7 ± 5.3 y, *vs. *28.8 ± 6.5 y (*p *= 0.85) in the non-attenders and 28.13 ± 6.7 y (*p *= 0.10) in the under-attenders. The youngest mother in this study was 14 y and the oldest 52 y. The study population was ethnically homogeneous.

Non-attenders and under-attenders of antenatal care were statistically significantly more often unmarried, smokers, and less often highly educated (Table 1.). Young age was more common in the group of non-attenders than average. Moreover, grand multiparity was more common in under-attenders than on average, as was alcohol use during pregnancy. Furthermore, the pregnancies of non-attenders and under-attenders of antenatal care were significantly more often (p < 0.001) complicated by placental abruption (4.44% and 6.28% vs. 0.70%, respectively) or chorio-amnionitis (4.83% and 9.66% vs. 1.37%, respectively), but no differences were found as regards other pregnancy complications.

**Table 1:** Occurrences of pregnancy risk factors and complications (%)in the study groups

	**Reference (6–18 visits) (*n *= 23137) %**	**No visits (*n *= 270) %**	**1–5 visits (*n *= 207) %**
**Pregnancy risk factors**			

Age < 18 years	0.63	1.85 **	0.97
Age > 35 years	11.03	14.07	12.56
Unmarried	17.48	30.74 ***	33.33 ***
Unemployed	16.97	16.67	16.43
Education			
High	24.74	18.51 *	16.43 **
Average	45.71	34.07 ***	36.23 **
Low	21.70	22.96	20.77
Primiparity	40.52	43.70	41.55
Multiparity	0.41	0.37	1.45 *
Prior miscarriage	9.78	16.67	17.88
Prior induced abortion	9.78	7.78	12.56
Prior fetal death	1.79	1.48	2.42
Overweight	19.04	22.17	18.93
History of infertility	6.30	5.93	4.35
Smoking	5.88	8.52	13.04 ***
Alcohol consumption	3.45	3.70	6.76 **
Chronic illness	5.13	4.44	5.80
Diabetes	2.25	1.85	1.93
Chronic hypertension	1.28	1.11	0.97

**Pregnancy complications**			

Prolonged gravidity	3.77	4.44	2.08
Low haemoglobin	1.53	0.74	2.90
Pre-eclampsia	2.76	3.72	3.86
Chorio-amnionitis	1.37	4.83 ***	9.66 ***
Placental abruption	0.70	4.44 ***	6.28 ***
Placenta previa	0.41	0.37	0.48
Meconium-stained AF	10.56	10.98	13.13
Abnormal FHR during delivery	16.76	19.02	15.20

AF = amniotic fluid; FHR = fetal heart rate; *p < 0.05, **p < 0.01, ***p < 0.001

The mean birth weight (± standard deviation) of newborns was 3503 ± 617 g in the women with 6–18 antenatal visits, 3014 ± 1033 g in the non-attenders and 2704 ± 1156 g in the under-attenders. Accordingly, the newborns of non-attenders and under-attenders of antenatal care weighed 489 and 799 g less, respectively, than the newborns of mothers with an average number of antenatal visits, these differences being statistically significant (*p *< 0.0001).

Table 2 shows unadjusted odds ratios (OR:s) and the results of logistic regression analyses, the adjusted odds ratios of adverse pregnancy outcome in the study groups. Before these analyses, non-attenders and under-attenders statistically significantly more often had low 5-minute Apgar scores, preterm births and low birth weight infants. Additionally, the risk of fetal death was high in both the study groups, as well as was the risk of neonatal death. Controlling for confounding factors in logistic regression analyses did not diminish the observed risks.

**Table 2:** Ocurrences of pregnancy outcomes (%) in the study groups and relative risks (OR) compared with the reference group

Outcome	Study group^a^	%	Unadjusted OR	95% CI	Adjusted OR*	95% CI
SGA	Reference	9.29				
	0	8.15	0.87	0.56–1.34	0.97	0.62–1.52
	1–5	9.66	1.04	0.66–1.66	0.97	0.60–1.55
Preterm delivery	Reference	6.51				
(< 37 weeks)	0	25.22	4.84	3.57–6.57	4.60	3.35–6.31
	1–5	39.58	8.51	6.39–11.35	8.58	6.32–11.64
Low birth weight	Reference	4.73				
(< 2500 g)	0	21.85	5.63	4.19–7.57	5.46	3.90–7.65
	1–5	33.01	9.86	7.33–13.26	9.18	6.65–12.68
Admission to NICU	Reference	7.76				
	0	9.26	1.21	0.80–1.83	1.18	0.75–1.86
	1–5	8.70	1.13	0.70–1.84	1.14	0.69–1.86
Low Apgar score (< 7) 1 min	Reference	4.83				
	0	18.89	4.59	3.36–6.26	4.52	3.20–6.38
	1–5	25.60	6.78	4.93–9.31	6.90	4.94–9.64
Low Apgar score (< 7) 5 min	Reference	1.82				
	0	11.11	6.74	4.56–9.98	5.98	3.80–9.40
	1–5	14.98	9.50	6.41–14.09	9.70	3.40–14.71
Foetal venous pH < 7.15 at birth	Reference	1.22				
	0	3.70	3.12	1.64–5.93	1.13	0.28–4.62
	1–5	1.93	1.60	0.59–4.33	3.71	1.48–9.28
Caesarean section	Reference	16.22				
	0	23.33	1.57	1.18–2.09	1.68	1.23–2.29
	1–5	21.26	1.39	1.0–1.95	1.50	1.06–2.12
Fetal death	Reference	0.26				
	0	2.59	5.76	2.50–13.27	5.19	2.04–13.22
	1–5	4.83	12.86	6.59–25.07	12.05	5.95–24.4
Neonatal death	Reference	0.25				
	0	2.59	10.41	4.71–23.01	8.66	3.59–20.86
	1–5	2.42	9.68	3.85–24.38	10.03	3.85–26.13

Adjusted for: Age under 18 y, Age over 35 y, unmarried status, smoking during pregnancy, using alcohol during pregnancy, educational level, primiparity, multiparity, prolonged gravidity, chronic illness and diabetes

SGA = small for gestational age; NICU = neonatal intensive care unit

Table 3 summarizes the odds ratios of pregnancy risk factors and outcomes reported in earlier studies concerning inadequate maternity care, compared with the present data. The comparison suggests that when high frequencies of women receive insufficient antenatal care, the magnitude of associating risks is diluted.

**Table 3:** Pregnancy risk factors and relative risks (OR, 95% CI) of adverse pregnancy outcomes of women with insufficient antenatal care

**Pregnancy risk factors**						
Study	[9]	[31]	[16]	[8]	[18]	Present study^1^
n, population	10382 two months national cohort, register data	85066 6 months hospital based cohort, clinical records (20 clinics)	21 722 Case-control, postpartum interview	17765 two months cohort of nine maternity units, clinical records	8065 one month cohort of six hospitals, clinical records	23 614 twelve years hospital based register data
Country	Jamaica	France	Ten European countries	England and Wales	USA	Finland
Inadequate antenatal care	4%	1.1%	5.9%	7%	10.2%	1.8%
Age < 18	1.7 (1.2–2.2)3	2.8 (1.2–6.6)^2^	3.7 (2.7–5.1)	2.5 (2.0–3.0)	NA	1.29 (0.60–2.74)
Unmarried	1.6 (1.1–2.2)	9.3 (6.0–14.3)^1^	3.1 (2.5–3.9)	NA	NA	1.88 (1.56–2.25)
Multiparous (> 4)	1.5 (0.9–2.4)	34.9 (15.7–77.8)^2^	4.3 (3.1–6.0)	NA	2.26 (1.76–2.90)	2.03 (0.74–5.54) 4
Unplanned pregnancy	2.8 (1.6–4.7)	NA	4.0 (3.3–4.7)	NA	NA	NA
No health insurance	NA	7.6 (2.2–26.8)^2^	2.7 (2.1–3.4)	NA	7.67 (5.96–9.86)	NA
Smoking	2.5 (1.8–3.4)	NA	NA	1.6 (1.4–1.9)2	NA	1.87(1.39–2.52)
Alcohol consumption	0.7 (0.5–0.9)	NA	NA	NA	NA	1.48(0.98–2.25)

**Pregnancy outcome**						

Study	[6]	[32]	[31]	[18]	Present study^1^	
*n*	344	57108	85066	8065	23 614	
*n*, population	Register based study (religional minority, national obstetric statistics)	One year national cohort, register data	6 months hospital based cohort, clinical records (20 clinics)	one month cohort of six hospitals, clinical records	Mothers Under-attending antenatal care	Mothers Not-attending antenatal care
Country	USA	Finland	France	USA	Finland	
Preterm birth	NA	2.21 (1.95–2.51)^2^	5.8 (3.2–10.5)^2^	3.23 (2.62–3.99)	6.50 (4.71–8.99)^2^	4.60 (3.35–6.31)^2^
Low birth weight	NA	2.05 (1.74–2.41)^2^	2.6 (1.5–4.4)^2^	2.20 (1.72–2.79)	6.62 (4.50–9.32)^2^	5.46 (3.90–7.65)^2^
Admission to NICU	NA	1.56 (1.24–1.98)^2^	2.8 (1.9–4.1)	NA	0.87 (0.52–1.44)^2^	1.18 (0.75–1.86)^2^
Fetal death	3.6 (1.8–6.3)	NA	NA	NA	7.75 (3.65–16.46)^2^	5.19 (2.04–13.22)2
Perinatal death	2.7 (1.6–4.2)	1.87 (1.34–2.62)^2^	NA	NA	NA	NA
Neonatal death				3.63 (2.23–5.91)	6.16 (4.70–9.32)^2^	8.66 (3.59–20.86)2

^1^Pooled, under-attending and non-attending ^2^OR adjusted for confounding factors found in the study; ^3^age under 20 years; NA = not applicable; ^4^over 7 births; ^5^number of visits relative to gestational length

## Discussion

Regardless of easily accessible and high quality maternity care a small minority of pregnant women chose not to use it. The outcome of their pregnancies was poor, although delivery took place in hospital, in conditions of modern obstetric care. Specifically, the risk of placental abruption, intrauterine infections, preterm birth, low birth weight and even intrauterine fetal death and neonatal death were found to be statistically higher than in the general obstetric population who attended routine antenatal care. Clinically, under-attending antenatal care appeared to be a significant contributor to low birth weight, and this association was chiefly the result of preterm delivery, not to growth restriction.

Only a few studies concerning pregnancy outcome in women under-attending antenatal care have been published (Table 3). Overall, the present study showed similar outcomes as in earlier studies, although the magnitude of risk appears to vary substantially in different settings depending on the antenatal care system and degree of low attendance. Comparison with the results of prior studies also suggests that the magnitude of the risk may be diluted in settings with a high frequency of women receiving inadequate antenatal care. Accordingly, definitions used for inadequate antenatal care vary from late attendance to a reduced total number of visits. Although the risk profile of women in the present study resembles that published earlier [8,9,16-20], socio-demographic and health behavioral risk factors appeared to play a less significant role in our country than elsewhere, probably because the care is offered free of charge and is readily and easily accessible to all women. Interestingly, a substantial proportion (52.5%) of non-attenders and under-attenders were of high or average educational level in the present study.

Surprisingly, factors that have been reported to lead to higher concern and motivation to attend antenatal care were not under-presented in women not attending antenatal care: specifically, history of infertility treatment, miscarriage or fetal demise [7]. Furthermore, we found no differences between study groups in a number of the known risk factors associated with adverse health behavior and the lack of health consciousness during pregnancy, specifically unemployment [21], prior pregnancy terminations [22] and an overweight condition [23]. Accordingly, ethnicity of the mother, the nature of the antenatal care provider, health insurance and difficulties in accessibility of antenatal care, which have also been found to be important factors in the etiology of under-attending antenatal care [8], were not relevant in the present study.

Preterm birth is an extremely heterogeneous index by which to assess obstetric outcome, because it combines a number of intrinsic pathways resulting in the same endpoint [24]. Efforts to isolate these pathways would benefit from studying the individual components. In that regard, the substantially high incidence of placental abruption found in the present study is partly explained by smoking during pregnancy [25,26], but it also raises a hypothesis of trauma and domestic violence as possible explanations for adverse pregnancy outcome and a reduction in the uptake of services [27]. Amnionitis and neonatal deaths have been reported to be associated with a number of underlying risks, such as experienced violence during pregnancy and changing partners [28-30].

Overall, as failure to attend antenatal care is very rare in Finland, the underlying reasons probably varied and it can be assumed that women choosing to self exclude themselves from antenatal care have some serious but still poorly recognized problems, and difficulties in their overall health behavior. This could partly explain the higher numbers of adverse pregnancy outcomes found in this study compared with earlier studies [6,18,31,32].

A strength of the present study is that we had the opportunity to assess the effects of maternal behavior and pre-pregnancy health on pregnancy outcomes, since the Kuopio University Hospital birth registry contains comprehensive data on these variables. A possible limitation of this data from a tertiary level perinatal centre in this area is that some adverse outcomes may be overly present, but this is probably the case particularly in the reference population. Furthermore, estimation of gestational age in pregnancies with a lack of antenatal care may not be as accurate as normally. However, inaccuracy in assessing the risk of prematurity was overcome by the high percentage of low birth weight infants and the lower mean birth weights found in women lacking in antenatal care. The high amount of preterm births (39.6%) in the group of women having only 1–5 visits at antenatal care is partly the explanation for few visits. However, the outcome was very much alike in the group of women totally lacking antenatal care. Accordingly, compared with a prior Finnish study [32], the risks of prematurity, NICU-treatments and perinatal death were higher in our study, probably since we did not adjust the number of antenatal visits according to gestational age. Moreover, the background data for women not attending antenatal care was collected at the time of the birth, may have underreporting as a source of error, depending on the pregnancy outcome.

Furthermore, by definition, distinguishing confounding factors from mediating factors as regards lack of antenatal care and adverse pregnancy outcomes is difficult. However, pregnancy outcome measures in the groups studied were compared both before and after adjusting for these factors, to overcome the difficulty brought about by either the confounding or mediating roles of the known obstetric risks significantly associated with under-attending antenatal care.

The role of domestic violence as an etiological factor needs future investigation. Other possible explanations that require future research are psychiatric disorders and ideological reasons for refusing antenatal care (naturalism, avoidance of technology, religion). Moreover, it would be interesting to investigate the children of the women who excluded themselves from maternity care.

## Conclusion

In conclusion, the results of this study highlight to two things: First, it is important to recognize women under-attending antenatal care during pregnancy as high-risk obstetric patients who need extra surveillance during delivery, support when going home with the newborn, and probably support in responsible health behavior in the future. Second, our results underline the beneficial role of maternity care not only as regards recognizing and treating pregnancy pathology, but also in terms of a resource of health education and an environment for confidential handling of sensitive issues, such as unwanted pregnancy or domestic violence.

## Competing interests

The author(s) declare that they have no competing interests.

## Authors' contributions

All authors (KR, NH, and SH) participated in designing the study, analyzing the results and writing the manuscript, KR coordinated the study. All authors read and approved the final manuscript.

## Pre-publication history

The pre-publication history for this paper can be accessed here:


